# Sympathetic Nerve Block in Lymphedema Treatment: A Systematic Review

**DOI:** 10.7759/cureus.5700

**Published:** 2019-09-19

**Authors:** Antonio J Forte, Daniel Boczar, Maria T Huayllani, Xiaona Lu, Sarah A McLaughlin

**Affiliations:** 1 Plastic Surgery, Mayo Clinic Florida - Robert D. and Patricia E. Kern Center for the Science of Health Care Delivery, Jacksonville, USA; 2 Division of Plastic Surgery, Yale University, New Haven, USA; 3 Surgery, Mayo Clinic Florida - Robert D. and Patricia E. Kern Center for the Science of Health Care Delivery, Jacksonville, USA

**Keywords:** stellate ganglion block, breast cancer, lymphedema, treatment, plastic surgery, lower extremity, upper extremity, physical therapy, regional anesthesia

## Abstract

Although the sympathetic nerve system has been described as a modulator of lymphatic circulation, it has not been targeted in the treatment of lymphedema. We conducted a systematic review of publications assessing the use of sympathetic nerve block in lymphedema treatment. We hypothesized that sympathetic nerve block may be a promising treatment option for lymphedema patients. We conducted a comprehensive systematic review of the published literature on the use of sympathetic nerve block in lymphedema treatment using the PubMed database. Eligibility criteria excluded papers that reported other types of lymphedema treatment or any other anesthesiology procedure. Abstracts, presentations, reviews, and meta-analyses were also excluded. Extracted data included the year of study, country, author affiliation, type of study, patient characteristics, nerve block technique, and key findings. From 81 potential papers, eight studies fulfilled the eligibility criteria. All papers identified were clinical, reporting on a total of 187 patients. Sympathetic nerve block was proposed with local anesthetics, whether or not associated with triamcinolone. Treatment with a nerve block promoted lymphedema improvement expressed by decreased limb circumference and patient-reported outcomes. Large randomized clinical trials are still pending, but sympathetic nerve block seems to be a promising alternative for lymphedema patients who do not respond to conservative therapy.

## Introduction and background

Lymphedema is a chronic condition that affects five to six million people in the United States alone. In developed countries, lymphedema is mostly related to cancer treatment, having incidences as high as one in every six patients undergoing surgical treatment of a solid tumor [1]. Considering that lymphedema is still an incurable disease, studies on targeted therapies have attracted the attention of the scientific community.

It is well-accepted that the physiopathology behind lymphedema is associated with inflammation and fibrosis, where normal tissue is replaced by scar tissue [2-4]. Therefore, it is reasonable that most of the targeted therapies already proposed in the literature are related to inflammation modulation, such as Th2-inflammatory responses, and lymphangiogenic cytokines, such as vascular endothelial growth factor C (VEGF-C) [5-9]. Nonetheless, the translation of some of these therapies into clinical practice raises concern for metastasis in patients with a medical history of cancer [6].

The sympathetic nerve system has been described as a modulator of lymphatic circulation; however, it has not been a topic of debate in the literature about targeted therapies in the treatment of lymphedema [10-11]. Therefore, we conducted a systematic review of publications assessing the use of sympathetic nerve block in lymphedema treatment. We hypothesized that sympathetic nerve block may be a promising treatment for lymphedema patients.

## Review

Materials and methods

Search Strategy

Two reviewers (D.B., M.H.) conducted independent searches using the PubMed database without timeframe limitations. The initial search included title and abstract screening, followed by a full-text review. Disagreements regarding article identification and final selection for inclusion were resolved by another reviewer (A.J.F.). The search was done using the following keywords: (((((Anesthetics) OR Local Anesthetics) OR Autonomic Nerve Block) OR Stellate Ganglion)) AND ((Lymphedema) OR Breast Cancer Lymphedema). The bibliographies of the studies that fulfilled the study eligibility criteria were also examined, looking for articles not present in our initial search. This study followed the guidelines outlined in the preferred reporting items for systematic reviews and meta-analyses (PRISMA).

Selection Criteria

Eligibility criteria included studies reporting data from the use of sympathetic nerve block in the treatment of lymphedema. Therefore, we excluded papers that reported other types of lymphedema treatment or anesthesiology procedures. Abstracts, presentations, reviews, and meta-analyses were also excluded.

Data Extraction and Processing

Extracted data included the year of study, country, author affiliation, type of study, patient characteristics, nerve block technique, and key findings. Data were extracted from articles, tables, and figures by two reviewers (D.B., M.H.), with the accuracy of data entry confirmed by an additional reviewer (A.J.F.).

Results

Study Characteristics

**Figure 1:**
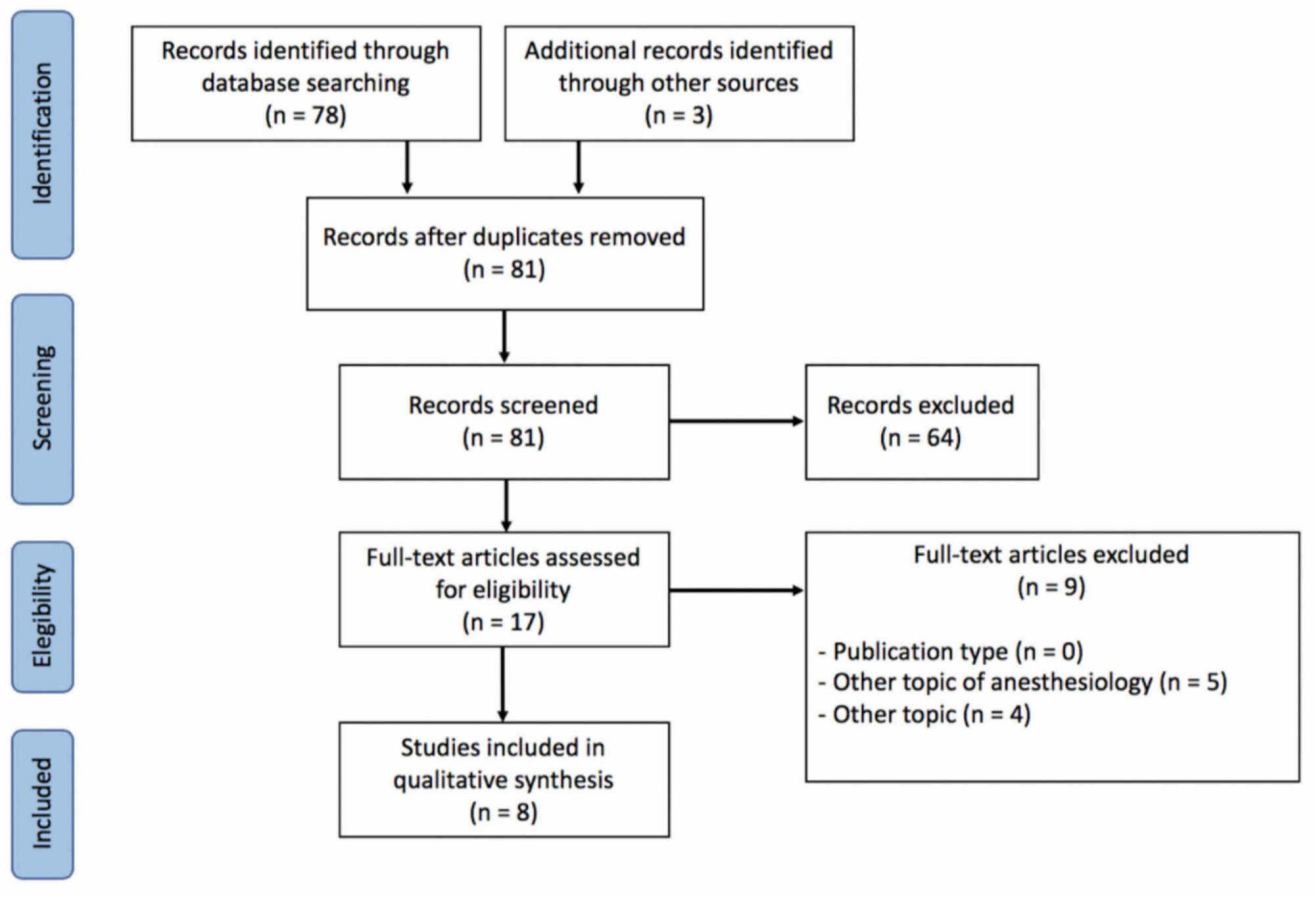
Preferred Reporting Items for Systematic Reviews and Meta-Analyses (PRISMA) Diagram

From 81 potential papers found in the literature, eight studies fulfilled the eligibility criteria (Figure 1, Table 1). While the first publication about sympathetic nerve block in lymphedema treatment was a case report in 1983 by authors from Sweden, most studies were conducted by groups in Asia. Interestingly, all authors were affiliated to the department of anesthesiology or rehabilitation medicine. Sympathetic nerve block was proposed with local anesthetics, whether or not associated with triamcinolone, at the lumbar spine (for lower extremity lymphedema) or at the stellate ganglion (for upper extremity lymphedema). All papers identified were clinical, reporting on a total of 187 patients. Lymphedema improvement was measured by authors through limb circumference and patient-reported outcomes. All papers identified had small sample sizes, and only two had control groups [12-13].

**Table 1:** Summary of Study Findings Abbreviations: BCRL, Breast Cancer-Related Lymphedema; LE, Lymphedema; SGS, Stellate Ganglion Blocks; CDT, Complex Decongestive Therapy; LSGB, Lumbar Sympathetic Ganglion Block

Author	Year	Country	Author affiliation	Study type	Patient	Procedure	LE improvement compared to patient baseline	Outcome compared to CTC
Swedborg et al. [14]	1983	Sweden	Department of Physical Medicine and Rehabilitation	Case report	1 patient, BCRL	SGB	Yes	-
Asai et al. [15]	2001	Japan	Department of Anesthesiology and Critical Care Medicine	Case report	1 patient, Cervical cancer; Bilateral lower limb lymphedema	Bilateral LSGB	Yes	-
Woo et al. [16]	2013	Korea	Department of Anesthesiology and Pain Medicine	Prospective clinical study	18 patients, Gynecological cancer; Lower limb lymphedema	LSGB	Yes	-
Choi et al. [17]	2015	Korea	Department of Anesthesiology and Pain Medicine	Retrospective study.	35 patients, BCRL	SGB	Yes	-
Jin Kim et al. [18]	2015	Korea	Department of Anesthesiology and Pain Medicine	Case series	2 patients; BCRL	SGB	Yes	-
Jeong-Gil Kim et al. [12]	2015	Korea	Department of Rehabilitation Medicine	Retrospective matched cohort study	60 patients, BCRL	SGB (30 patients) CDT (30 patients)	Yes	Both treatments improved LE. Difference between them was not statistically Significant.
Jae Hyeon Park et al. [19]	2015	Korea	Department of Rehabilitation Medicine	Double-blinded, randomized, controlled trial	32 patients, BCRL	SGB	Yes	-
Myung Woo Park et al. [13]	2019	Korea	Department of Rehabilitation Medicine	A randomized controlled trial	38 patients, BCRL	SGB (19 patients) CDT (19 patients)	Yes	Both treatments improved LE. Difference between them was not statistically Significant.

Case Reports and Case Series

The improvement of lymphedema following sympathetic nerve block has been described in two case reports and a case series. In 1983, Swedborg et al. were the first to describe a patient with breast cancer-related lymphedema who presented with a clinical improvement after a local sympathetic nerve block [14]. Jin Kim et al. described two patients with breast cancer-related lymphedema who saw a decrease in their affected limb’s circumference after the second stellate ganglion nerve block [18]. They conceptualize a series of blocks to prolong the effects [18]. Asai et al. described a 70-year-old patient with bilateral lower extremity lymphedema following surgical treatment for cervical cancer that “dramatically” improved after a sympathetic ganglion block [15]. Although these studies demonstrated the positive effects of sympathetic nerve block on lymphedema, conclusions were limited by the small number of patients and the absence of control groups.

Retrospective Studies

Retrospective studies published on the use of sympathetic nerve block in lymphedema treatment demonstrated promising outcomes. Choi et al. assessed patient-reported outcomes and the arm circumference of 35 patients with breast cancer-related lymphedema who underwent sympathetic nerve block [17]. At two months after the nerve block, there was a statistically significant improvement in arm circumference and patient-reported outcomes. The nerve block was effective in 65.7% of patients. Interestingly, the block was more effective in patients with high-stage lymphedema as compared to those with lower stages, and improvement was not correlated with the length of time the patient had lymphedema. Their conclusions were limited by the absence of a control group [17]. Kim et al. conducted a retrospective matched cohort study of 60 patients with breast cancer-related lymphedema [12]. Thirty patients underwent sympathetic nerve block while 30 underwent complex decongestive physiotherapy (standard treatment). Both groups were treated for two weeks. The nerve block was conducted three times, once every two weeks. Both groups had significant improvement in limb circumference compared to patient baseline. Interestingly, reduction in limb circumference was higher in the group treated with a nerve block, but the difference was not statistically significant [12].

Prospective Studies

Woo et al. conducted a prospective study of 18 patients with stage II gynecologic cancer-related lymphedema, refractory to treatment [16]. They demonstrated that lumbar sympathetic ganglion block decreased the circumference of affected limbs after three consecutive nerve blocks. Moreover, patients also reported a reduction in pain, tightness, and sensation of heaviness. Their study was limited by the small number of patients and the lack of a control group [16]. Jae Hyeon Park et al. conducted a double-blind, randomized, controlled trial, in which 32 patients with breast cancer-related lymphedema who underwent three consecutive sympathetic nerve blocks, one every two weeks [19]. They divided the patients into three groups: Group A received 0.5% bupivacaine 5 mL; Group B, 0.5% bupivacaine 4.5 mL with 20 mg of triamcinolone 0.5 mL; and Group C, 0.5% bupivacaine 4 mL with 40 mg of triamcinolone 1 mL. They demonstrated that sympathetic nerve block significantly decreased the measurements of the affected limbs. Interestingly, Group C obtained a statistically significant reduction in limb circumference as compared to patients in Group A [19]. Myung Woo Park et al. conducted a randomized control trial comparing the effects of a stellate ganglion block with complex decongestive therapy in 38 patients with breast cancer-related lymphedema [13]. They pointed out that both neural block and complex decongestive therapy decreased limb circumference as compared to the baseline. While they did not observe a statistically significant difference between the treatments, their study was limited by the small study population [13].

Discussion

Although the sympathetic nerve system modulation of the lymphatic system is relatively well-established, few authors have pursued this mechanism to propose targeted therapies in lymphedema treatment [10-1]. In this systematic literature review, we have shown that different authors have published positive clinical outcomes for sympathetic nerve blocks in the treatment of lymphedema. While all papers included in this study were limited by the small number of patients, promising results were found such as a decrease in limb circumference and improvement in patient-reported outcomes (eg, pain). Interestingly, sympathetic nerve block was associated with an improvement comparable with complex decongestive therapy (standard therapy) and was described as an effective therapy for patients with high-stage lymphedema, considered poor candidates for surgical treatments of lymphedema [17]. To our knowledge, this study is the first systematic literature review investigating the use of sympathetic nerve block in lymphedema treatment.

Sympathetic nerve block is a well-established treatment to manage patients with pain in the lower limbs [16]. Nerve blocks can be performed by anesthetic agents or electrical impulses [20-22]. From this literature review, we noticed that all efforts to propose sympathetic nerve block in lymphedema treatment used local anesthetics, such as bupivacaine, whether or not associated with triamcinolone. Interestingly, a prospective study conducted by Jae Hyeon Park et al. demonstrated that the association of bupivacaine with 40 mg of triamcinolone promoted better outcomes as compared to bupivacaine alone [19].

We do recognize the presence of limitations in this study common to systematic reviews, such as the potential for bias in interpreting the data reported in each study. Moreover, we only included studies published in the English language. In spite of these limitations, we feel that this systematic literature review summarized valuable data about the use of sympathetic nerve block in lymphedema treatment, which can guide future studies to advance the field. We encourage further studies on the topic with large prospective randomized clinical trials.

## Conclusions

The pooled publications investigating sympathetic nerve blocks in lymphedema treatment demonstrated positive outcomes. To date, all studies were clinical, using a local anesthetic to conduct the nerve block. Sympathetic nerve block promoted lymphedema improvement in affected limb circumference and patient-reported outcomes, such as pain, comparable to complex decongestive therapy. Large randomized clinical trials are still pending, but sympathetic nerve block seems to be a promising alternative for lymphedema patients who do not respond to conservative therapy.
